# Doppler ultrasonographic scan, gene expression and serum profile of immune, APPs and antioxidant markers in Egyptian buffalo–cows with clinical endometritis

**DOI:** 10.1038/s41598-024-56258-0

**Published:** 2024-03-08

**Authors:** Ahmed El-Sayed, Mohamed Refaai, Ahmed Ateya

**Affiliations:** 1 Department of Animal Health and Poultry, Animal and Poultry Production Division, Desert Research Center (DRC), Cairo, Egypt; 2 Diagnostic Imaging and Endoscopy Unit, Agriculture Research Centre, Animal Reproduction Research Institute, Giza, Egypt; 3 Department of Development of Animal Wealth, Faculty of Veterinary Medicine, Mansoura University, Mansoura, Egypt

**Keywords:** Buffaloes, Endometritis, Transrectal Doppler ultrasound, Gene expression, Single nucleotide polymorphisms, Antioxidants, Immunity, Immunology, Molecular biology, Zoology, Biomarkers, Cardiology, Health care, Risk factors

## Abstract

The objective of this study was to elaborate Doppler ultrasonographic scan, genetic resistance and serum profile of markers associated with endometritis susceptibility in Egyptian buffalo–cows. The enrolled animals were designed as; twenty five apparently healthy buffalo–cows considered as a control group and twenty five infected buffalo with endometritis. There were significant (*p* < 0.05) increased of cervical diameter, endometrium thickness, uterine horn diameter, TAMEAN, TAMAX and blood flow through middle uterine artery with significant decrease of PI and RI values in endometritis buffalo–cows. Gene expression levels were considerably higher in endometritis-affected buffaloes than in resistant ones for the genes *A2M*,* ADAMTS20*, *KCNT2*, *MAP3K4*, *MAPK14*, *FKBP5*, *FCAMR*, *TLR2*, *IRAK3*, *CCl2*, *EPHA4*, and *iNOS*. *The RXFP1*, *NDUFS5*, *TGF-β*, *SOD3*, *CAT*, and *GPX* genes were expressed at substantially lower levels in endometritis-affected buffaloes. The PCR-DNA sequence verdicts of healthy and affected buffaloes revealed differences in the SNPs in the amplified DNA bases related to endometritis for the investigated genes. However, *MAP3K4* elicited a monomorphic pattern. There was a significant decrease of red blood cells (RBCs) count, Hb and packed cell volume (PCV) with neutrophilia, lymphocytosis and monocytosis in endometritis group compared with healthy ones. The serum levels of Hp, SAA, Cp, IL-6, IL-10, TNF-α, NO and MDA were significantly (*P*˂0.05) increased, along with reduction of CAT, GPx, SOD and TAC in buffalo–cows with endometritis compared to healthy ones. The variability of Doppler ultrasonographic scan and studied genes alongside alterations in the serum profile of investigated markers could be a reference guide for limiting buffalo endometritis through selective breeding of natural resistant animals.

Although this species is typically raised in tough settings and exhibits limited reproductive and productive potentials, buffaloes are the primary source of high-quality meat and milk in Egypt and some other developing nations^1^. According to^2,3^, endometritis is characterized as a local inflammatory condition of the endometrium that results in large economic losses due to the delay in conception, increased artificial inseminations, expense of veterinary services, loss of the calf crop, and higher culling rate. Due to inadequate cleanliness, the shape of vulval lips, vaginal stimulation for milk let down, and wallowing behavior, buffalo–cows have a substantially greater prevalence rate of uterine infection than cows^4^. Uterine infection is one of the most significant reproductive issues in buffalo–cows, according to^5,6^.

In order to establish an accurate diagnosis and appropriate therapy, timing and diagnostic modalities are crucial^7^. Physical examination, ultrasonography, endometrial biopsies, cytology, and uterine culture are all necessary for the diagnosis of endometritis^8^. Although the development of transrectal ultrasound represented a significant advance in the assessment of uterine diseases^9^, it is not always simple to determine the precise diagnosis of endometritis. In order to analyze the reproductive system, Doppler ultrasound, a technique that can measure uterine blood flow, has recently been developed^10^ because endometrial alterations are linked to abnormalities in uterine blood flow and poor uterine vascular perfusion pattern^11^. The use of color Doppler ultrasonography to examine the correlations between intrauterine fluid buildup and uterine vascular perfusion is still relatively new, despite the growing popularity of Doppler in reproductive diagnostics.

Blood biochemical investigations give plenty of information about an animal's nutritional status, health, and wellbeing, therefore they can be used to assess the health of animals in general^12,13^. An estimate of the severity of the damage to the body's tissues could be made by observing a divergence of some blood values from their normal ranges^14^. An imbalance between the oxidant and antioxidant arms causes oxidative stress^15^. According to^16^, it has been reported to occur in bovine endometritis and is characterized by an increase in free radicals and nitric oxide. Sperm, ova, and embryogenesis are all affected by oxidative stress^17^.

A class of soluble proteins known as cytokines has the ability to control how cells and tissues function even at extremely low concentrations. They are created locally in response to stimuli, have a short half-life, and can act in an autocrine, exocrine, or endocrine manner^18^. Proinflammatory cytokine IL-6 is released by macrophages and T cells, and it is a strong pyrogen that causes the production of acute phase proteins (APP) by binding to receptors in hepatocytes^19^. An important antagonist of the Th1 type response is the anti-inflammatory cytokine IL-10^20^. To evaluate the innate immune system's response to infection or inflammation, serum cytokine levels can be evaluated.

The acute phase proteins (APP) are a class of blood proteins mostly made by the liver that are involved in the protection of infected animals from pathological harm, the maintenance of homeostasis, and the restriction of microbial growth in an antibody-independent way. Numerous pathogenic (including viral and non-infectious disorders) and physiological (including diet, age, sex, pregnancy, breastfeeding, and environmental variables) factors have an impact on APPs levels^21^. Due to the fact that APP levels are correlated with illness severity, they may also be employed as prognostic indicators and as measures of flock health^22^. According to^23^, Acute phase reactants can be classified as positive or negative, depending on their serum concentrations during inflammation. Positive acute phase reactants are upregulated, and their concentrations increase during inflammation. Negative acute phase reactants are downregulated, and their concentrations decrease during inflammation. Positive acute phase reactants include procalcitonin, C-reactive protein, ferritin, fibrinogen, hepcidin, and serum amyloid A. Negative acute phase reactants include albumin, prealbumin, transferrin, retinol-binding protein, and antithrombin. Haptoglobin (Hp) is the main APP in ruminants, and serum amyloid A (SAA) is among the positive APPs that see a rise throughout the AP response^24^.

The advanced molecular genetic techniques could help as adjunct to control the disease by improving animal health^25^. Several genetic markers, mostly single nucleotide polymorphisms (SNPs), have been successfully identified for disease susceptibility/resistance in livestock^26^. This suggests that there are variations between host genomes in the degree of susceptibility/resistance to the disease^27^. It is hypothesized that alterations in the hemodynamics of the uterus tissue and arteries may happen in buffalo cows presenting endometritis in the puerperal. Thus, the use of more than one diagnostic technique may help in the identification of uterine diseases. So far, there is limited information about the metabolic, immunological, antioxidant alterations; SNPs and gene expression associated with buffalo cow's endometritis. Therefore, the aim of the present study was to validate the diagnostic use of Doppler ultrasonography and exploring SNPs, gene expression and serum profile of APPs, immune and antioxidant markers of endometritis in buffalo cows.

## Results

### Clinical findings

There was a significant (*P* < 0.05) increase of body temperature, pulse and respiratory rates (39.9 ± 0.03 °C, 67.6 ± 4.3Beats/min and 31 ± 0.5Breaths/min), respectively in endometritis group in relation to control group (38.1 ± 0.2 °C, 48.6 ± 1.8Beats/min and 22 ± 1.1Breaths/min), respectively (Table 1).

**Table 1:** Changes in temperature, pulse and respiratory rates, in control (N = 25) and endometritis (N = 25) buffalo cows (mean ± SE).

Parameters	Control buffalo–cow	Endometritis buffalo–cow	*p* value
Temperature (°C)	38.1 ± 0.2	39.9 ± 0.03*	0.001
Pulse rate (Beats/min)	48.6 ± 1.8	67.6 ± 4.3*	0.01
Respiratory rate (Breaths/min)	22 ± 1.1	31 ± 0.5*	0.002

*Statistically significant when *P* < 0.05.

### Ultrasonographic and Doppler findings

**Figure 1:**
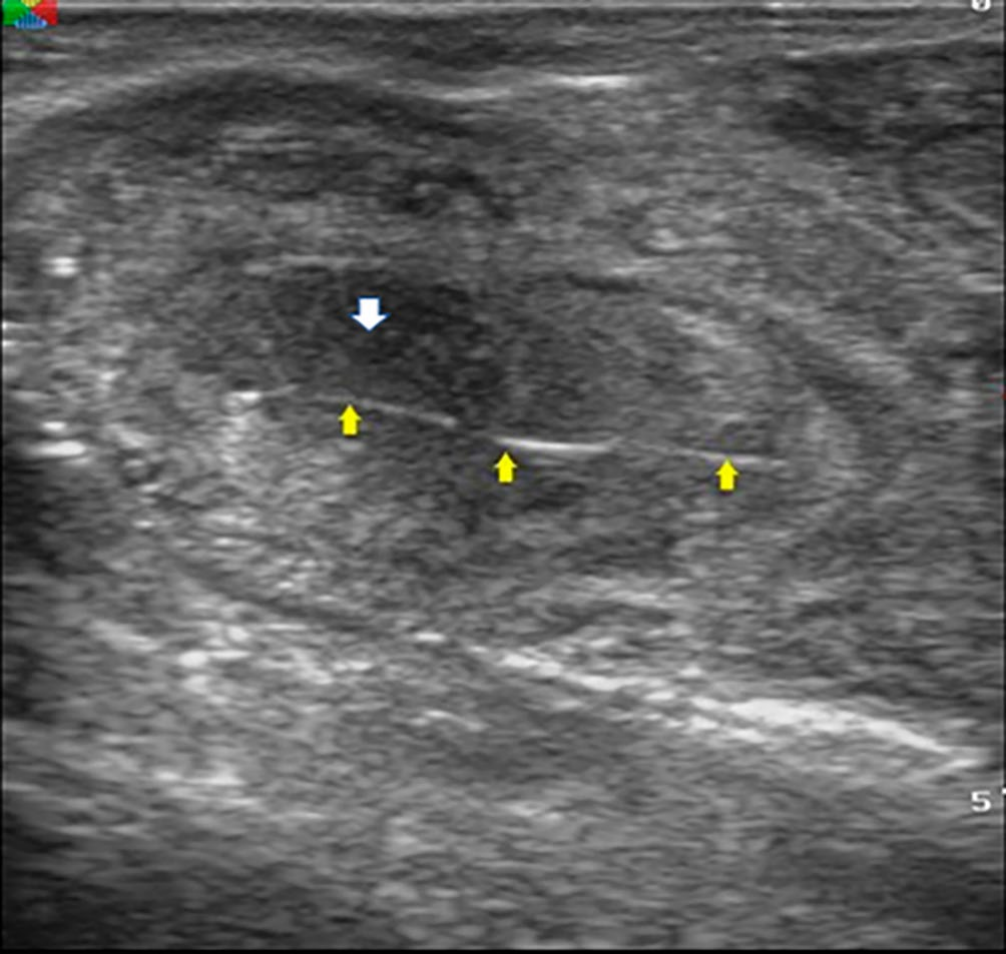
Ultrasonographic images of endometritis in buffalo cow characterized by a distended lumen filled to a varying degree with partially echogenic, ‘snowy’ patches (yellow arrow) and intrauterine heterogeneous content (white arrow).

**Figure 2:**
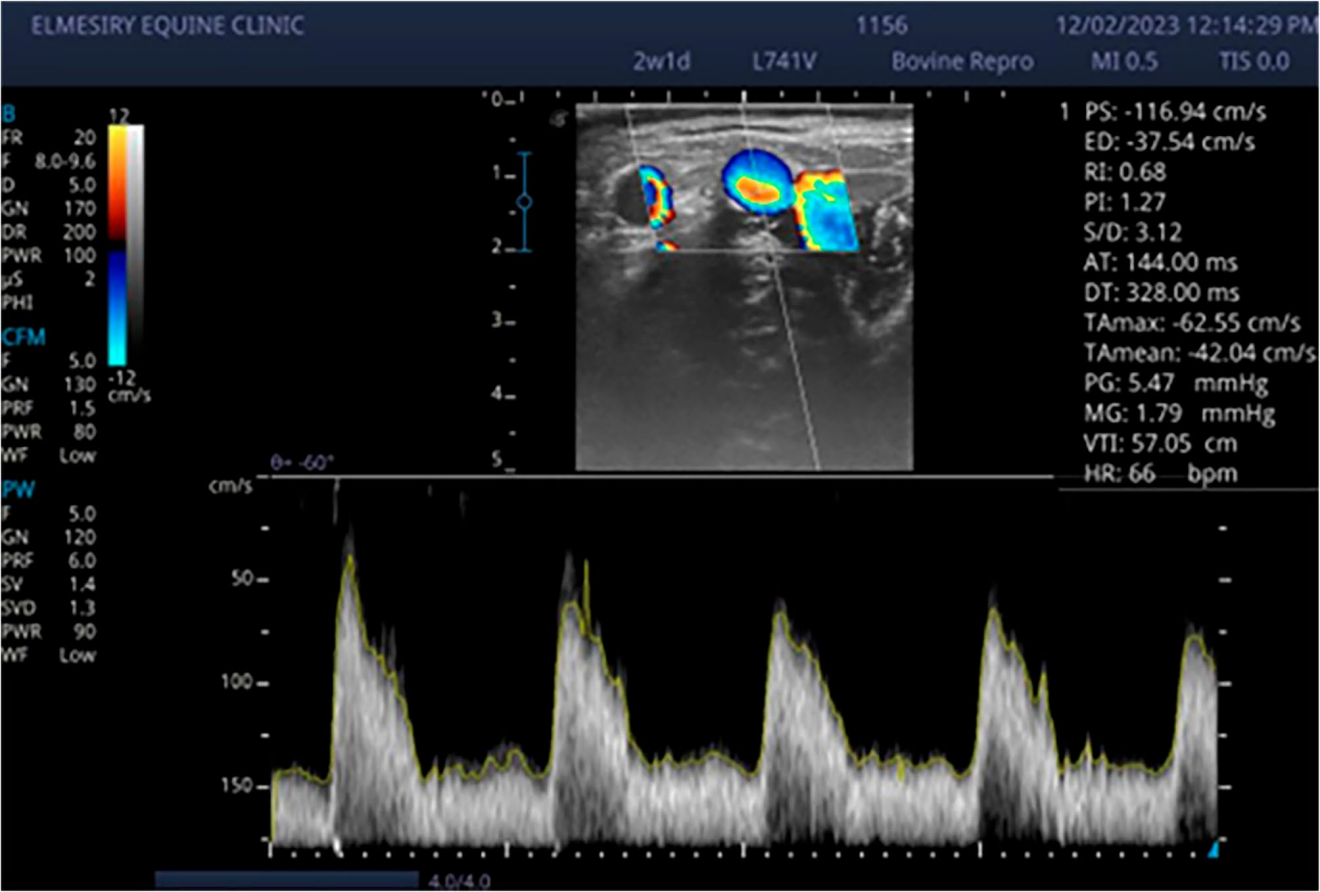
Doppler sonogram images illustrate waveform of at mid‐estrus buffalo cow; Waveforms diagnosed with clinical endometritis characterized by low RI and PI with significant (*p* < 0.05) increased of TAMEAN (cm/sec) and TAMAX (cm/sec).

The normal uterus at the time of estrus showed a distinct folding of the endometrium; generally, images of endometritis were characterized by a distended lumen filled to a varying degree with partially echogenic, ‘snowy’ patches and intrauterine heterogeneous content (Fig. 1). There were significant (*p* < 0.05) increased of cervical diameter, endometrium thickness, and uterine horn diameter between endometritis buffalo–cow and control ones (Table 2). There were significant (*p* < 0.05) decreased of PI and RI values with significant (*p* < 0.05) increased of TAMEAN, TAMAX and transverse diameter of middle uterine artery in endometritis buffalo–cows (Table 2 and Fig. 2). Also, significantly higher (*p* < 0.05) blood flow through middle uterine artery, that was BFV‐TAMAX and BFV‐TAMEAN was evident in endometritis buffalo–cow as compared to normal ones (Table 2).

**Table 2:** Transrectal ultrasonographic findings and Blood flow parameters in control (N = 25) and endometritis buffalo–cow (N = 25).

Parameters	Normal buffalo	Endometritis buffalo	*P* values
Cervical diameter (cm)	3 ± 0.01	3.6 ± 0.0.04*	0.001
Uterine horn diameter (cm)	2.2 ± 0.05	2.5 ± 0.01*	0.006
Endometrium thickness (cm)	0.65 ± 0.03	0.92 ± 0.02*	0.001
Pulsatility index	2.5 ± 0.2	1.4 ± 0.1*	0.01
Resistance index	0.88 ± 0.06	0.65 ± 0.02*	0.02
TAMAX (cm/sec)	28.7 ± 2.1	47 ± 2.1	0.004
TAMEAN (cm/sec)	15.8 ± 1.6	29.4 ± 2*	0.007
Diameter of the MUA (cm)	0.71 ± 0.03	0.89 ± 0.005	0.007
Blood flow volume‐ TAMAX (ml/min)	6.9 ± 1	17.4 ± 0.9*	0.002
Blood flow volume‐ TAMEAN (ml/min)	3.8 ± 0.5	10.9 ± 0.7*	0.001

*TAMAX* The time average maximum velocity, *TAMEAN* Time average mean velocity, *MUA* Middle uterine artery.

*Statistically significant when *P* < 0.05.

### Patterns for transcript levels of immune, metabolic, and antioxidant indicators

**Figure 3:**
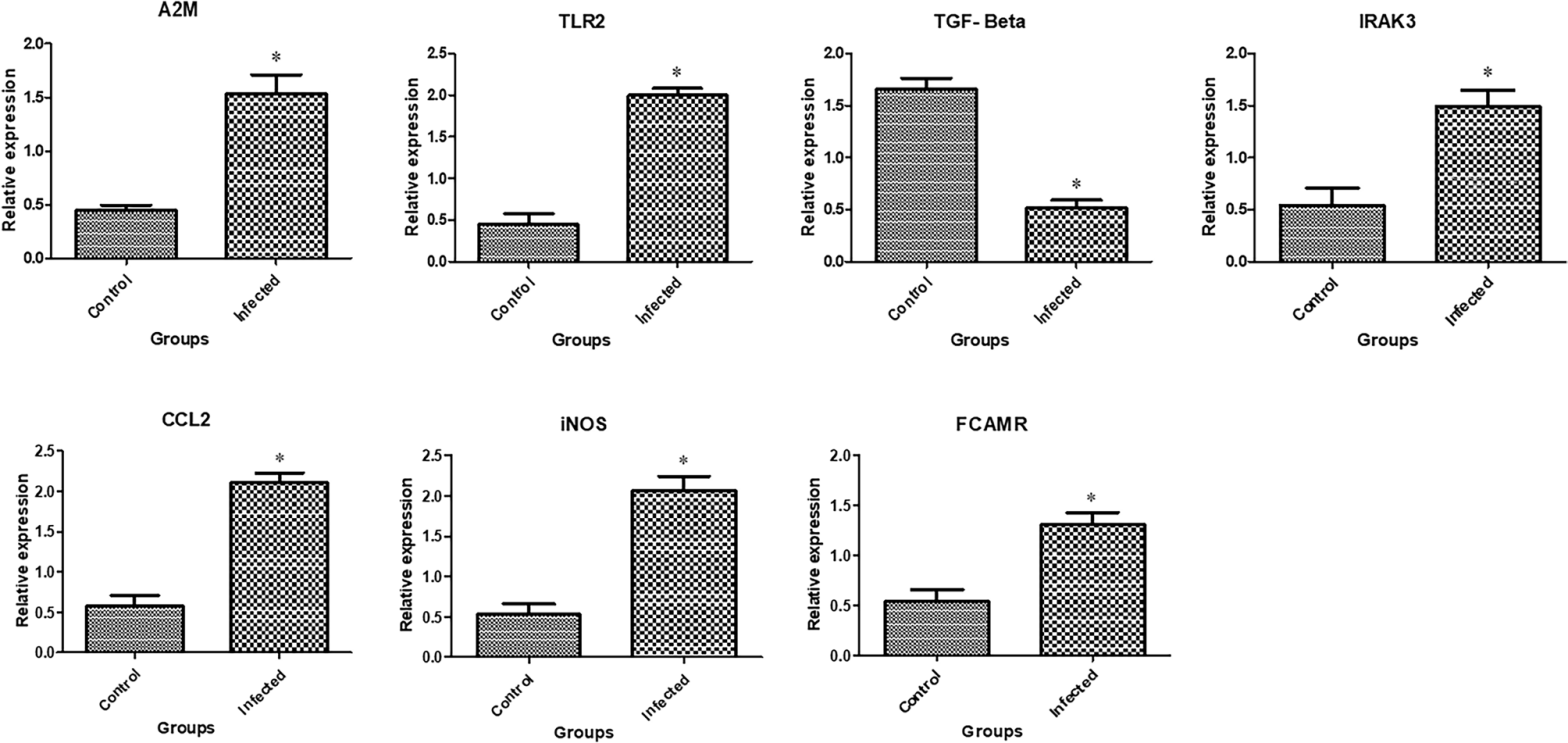
Different immune gene transcript levels between normal and endometritis affected buffaloes. The symbol *denotes significance when *p* < 0.05.

**Figure 4:**
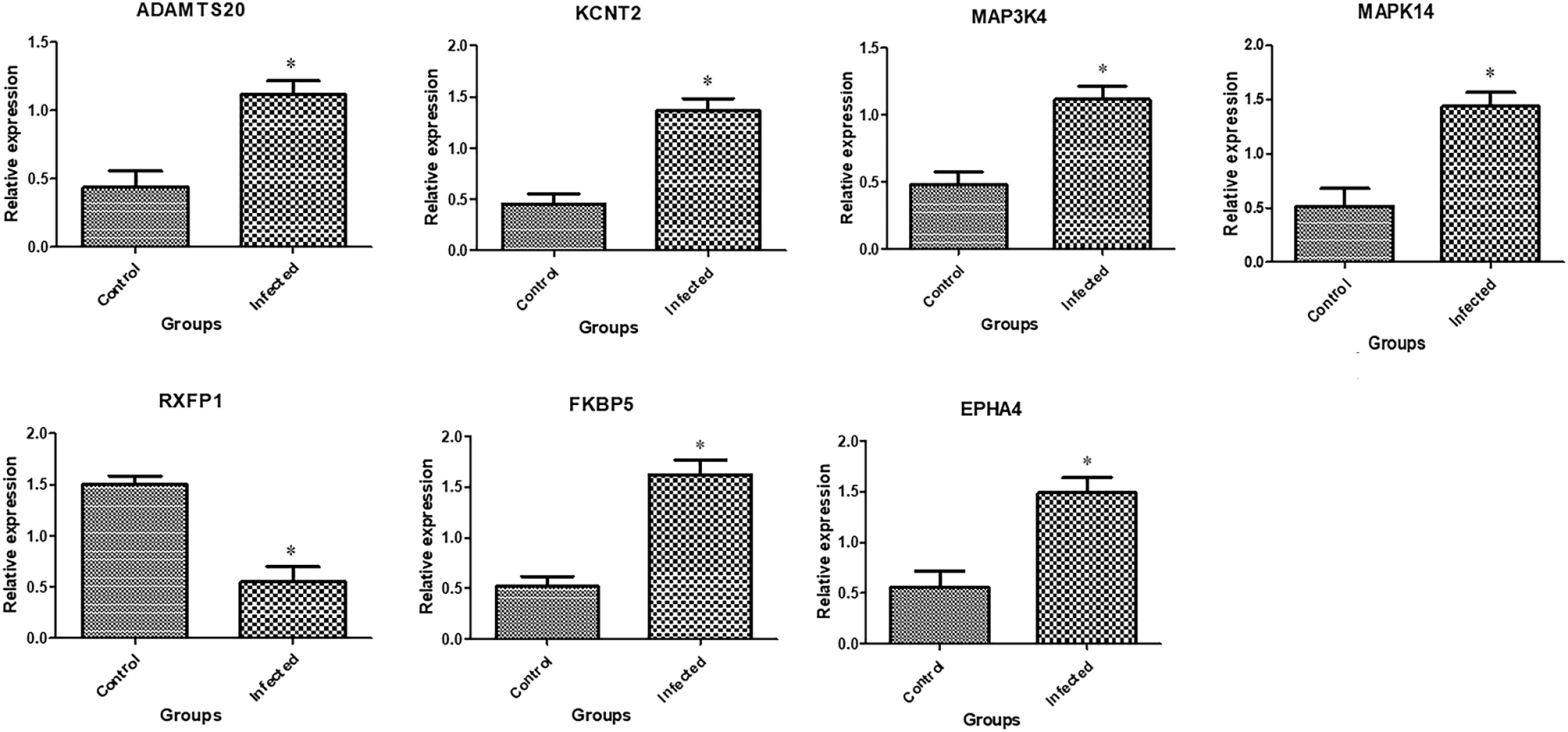
Different metabolic gene transcript levels between normal and endometritis affected buffaloes. The symbol *denotes significance when *p* < 0.05.

**Figure 5:**
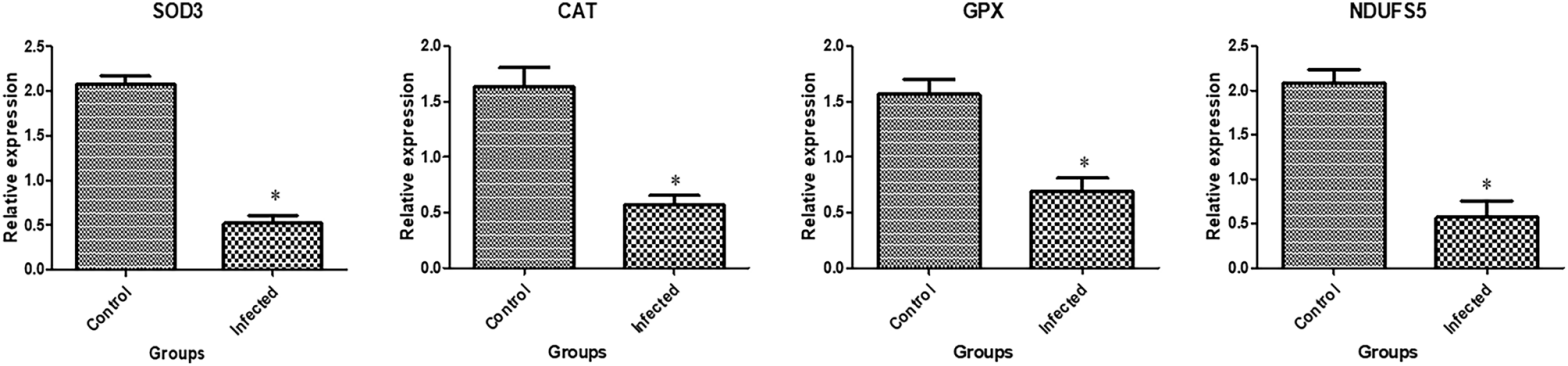
Different antioxidant gene transcript levels between normal and endometritis affected buffaloes. The symbol *denotes significance when *p* < 0.05.

In Figs. 3, 4, and 5 the transcript profiles for the assessed immune, metabolic, and antioxidant indicators are displayed. Gene expression levels were considerably higher in endometritis-affected buffaloes than in resistant ones for the genes *A2M*, *TLR2*, *IRAK3*, *CCl2*, *FCAMR*, *iNOS*, *ADAMTS20*, *KCNT2*, *MAP3K4*, *MAPK14*, *FKBP5,* and* EPHA4*. *The RXFP1*, *NDUFS5*, *TGF-β*, *SOD3*, *CAT*, and *GPX* genes were expressed at substantially lower levels in endometritis-affected buffaloes.

### Genetic polymorphisms of immune, metabolic and antioxidant genes

The PCR-DNA sequence verdicts of healthy and affected buffaloes revealed differences in the SNPs in the amplified DNA bases related to endometritis for the *A2M* (304-bp), *TLR2* (224-bp), *TGF-β* (420-bp), *IRAK3* (310-bp), *CCl2* (356-bp), *FCAMR* (330-bp), *iNOS* (445-bp), *ADAMTS20* (369-bp), *KCNT2* (420-bp), *MAPK14* (300-bp), *FKBP5* (345-bp), *RXFP1*(360-bp),* EPHA4* (420-bp), *SOD3* (360-bp), *CAT* (362-bp), *GPX* (287-bp), and *NDUFS5* (328-bp) genes. However, *MAP3K4* (450-bp) elicited a monomorphic pattern. All the discovered SNPs were approved using the DNA sequence differences between immune, metabolic, and antioxidant markers investigated in the researched buffaloes and the reference gene sequences obtained from GenBank. The exonic region changes were present in all of the immune, metabolic, and antioxidant markers under investigation, causing coding DNA sequence alterations in the affected buffaloes compared to healthy ones (Table 3).

**Table 3:** Distribution of SNPs, type of mutation in immune and antioxidant genes in healthy and endometritis affected buffaloes.

Gene	SNPs	Healthy	Mastitis	Total	Type of mutation	Amino acid number and type
*A2M*	C90T	0/25	14/25	14/50	Synonymous	30 V
	A242G	0/25	20/25	20/50	Non-synonymous	81 H to R
*TLR2*	G93A	8/25	0/25	8/50	Synonymous	31 T
*TGF-β*	T50C	0/25	21/25	21/50	Non-synonymous	17 I to T
	T372G	0/25	17/25	17/50	Non-synonymous	124 D to E
*IRAK3*	C73T	13/25	0/25	13/50	Non-synonymous	25 P to S
*CCL2*	G177A	0/25	9/25	9/50	Non-synonymous	59 M to I
	C222T	17/25	0/25	17/50	Synonymous	74 P
*FCAMR*	G178C	0/25	13/25	13/50	Non-synonymous	60 D to H
*iNOS*	T203C	0/25	14/25	14/50	Non-synonymous	68 L to P
*ADAMTS20*	A319C	19/25	0/25	19/50	Non-synonymous	107 L to I
*KCNT2*	T50C	0/25	23/25	23/50	Non-synonymous	17 S to F
	A226T	0/25	11/25	11/50	Non-synonymous	76 T to S
*MAP3K4*	G46A	14/25	0/25	14/50	Non-synonymous	16 V to I
	T159C	16/25	0/25	16/50	Synonymous	53 T
*RXFP1*	T219C	18/25	0/25	18/50	Synonymous	73 N
*FKBP5*	A227G	20/25	0/25	20/50	Non-synonymous	76 N to S
*EPHA4*	A31G	9/25	0/25	9/50	Non-synonymous	11 T to A
	T129G	12/25	0/25	12/50	Non-synonymous	43 S to R
*SOD3*	T117G	0/25	11/25	11/50	Synonymous	59 G
*CAT*	T175A	22/25	0/25	22/50	Non-synonymous	59 L to M
	A340G	0/25	18/25	18/50	Non-synonymous	114 M to V
*GPX*	G90T	0/25	13/25	13/50	Synonymous	30 A
*NDUFS5*	C179T	0/25	14/25	14/50	Non-synonymous	60 A to V

*A2M* alpha-2-macroglobulin, *TLR2* Toll-like receptor 2, *TGF-β* Transforming growth factor beta, *IRAK3* Interleukin 1 receptor associated kinase 3, *CCL2* C–C Motif Chemokine Ligand 2, *FCAMR* Fc alpha and Mu receptor, i*NOS* Inducible nitric oxide synthase,* ADAMTS20* ADAM metallopeptidase with thrombospondin type 1 motif 20, *KCNT2* Potassium sodium-activated channel subfamily T member 2, *MAP3K4* Mitogen-activated protein kinase kinase kinase 4, *MAPK14* Mitogen-activated protein kinase 14, *FKBP5* FKBP prolyl isomerase 5, *RXFP1* Relaxin family peptide receptor 1, *EPHA4* Ephrin type-A receptor 4, *SOD3* Superoxide dismutase 3, *CAT* Catalase, *GPX* Glutathione peroxidase, and *NDUFS5* NADH:ubiquinone oxidoreductase subunit s5. *A* Alanine, *D* Aspartic acid, *E* Glutamic acid, *F* Phenylalanine, *G* Glycine, *H* Histidine, *I* Isoleucine, *L* Leucine, *M* Methionine, *N* Asparagine, *P* Proline, *R* Argnine, *S* Serine, *T* Threonine, *V* Valine.

### Hematological and biochemical profile

Hematologically, Red blood cells (RBCs) count, Hb and packed cell volume (PCV) were significantly (*P*˂0.05) decreased in endometritis group compared with healthy ones; While leukogram revealed enhanced cellular immunity represented by neutrophilia, lymphocytosis and monocytosis (*P*˂0.05) in endometritis buffalo–cow; however no significant change were observed in values of MCV, MCH and MCHC in both groups (Table 4). Concerning the APPs and cytokines, the serum levels of Hp, SAA, Cp, IL-6, IL-10 and TNF-α were significantly (*P*˂0.05) increased in buffalo–cows with endometritis compared to healthy ones. With respect to changes in antioxidant/oxidative stress biomarkers, CAT, GPx, NO, SOD and TAC showed a significant decrease associated with a significant increase of MDA in buffalo–cows with endometritis compared to healthy group (Table 5).

**Table 4:** Some hematological parameters of control (N = 25) and endometritis (N = 25) buffalo cows.

Parameters	Normal buffalo	Endometritis buffalo	*P* values
RBC (× 10^12^/L)	5.2 ± 0.2	3.9 ± 0.0.1*	0.01
Hb (g/dl)	10.8 ± 0.4	9 ± 0.2*	0.02
PCV%	32.6 ± 1.7	24.3 ± 0.8*	0.01
MCV (fL)	50.2 ± 0.9	51.6 ± 2.6	0.65
MCH (pg)	17.2 ± 0.14	17.3 ± 0.14	0.65
MCHC (g/dl)	34.6 ± 0.7	35.2 ± 0.4	0.52
WBC(× 10^9^/L)	6.8 ± 0.4	10.6 ± 0.06*	0.001
Lymphocyte (× 10^9^/L)	1.6 ± 0.2	2.9 ± 0.0.4*	0.04
Monocyte (× 10^9^/L)	0.6 ± 0.02	0.9 ± 0.08*	0.02
Neutrophil (× 10^9^/L)	3.6 ± 0.12	7 ± 0.7*	0.01

*RBC* Erythrocytes count, *Hb* Hemoglobin, *PCV* Packed cell volume, *MCV* mean corpuscular volume, *MCH* mean corpuscular hemoglobin, *MCHC* Mean corpuscular hemoglobin concentration, *WBC* total leukocytes count.

*Statistically significant when *P* < 0.05.

**Table 5:** Serum acute phase proteins, immunological and oxidant/antioxidant values in control (N = 25) and endometritis buffalo–cow (N = 25) (Mean ± SE).

Parameters	Normal buffalo	Endometritis buffalo	*P* values
Haptoglobin (ng/ml)	58.6 ± 0.88	87.6 ± 2*	0.002
Serum amyloid A (mg/l)	4.9 ± 0.08	9.6 ± 0.17*	0.001
Ceruloplasmin (mg/dl)	22 ± 3.4	46.6 ± 3.2*	0.007
Interleukin 6 (pg/ml)	25.6 ± 3.1	85.6 ± 6.3*	0.004
Interleukin 10 (pg/ml)	27.3 ± 3.9	98 ± 8.5*	0.006
TNF-α (pg/mL)	42 ± 5.2	132 ± 16.7*	0.02
Catalase (U/l)	36 ± 0.57	16.3 ± 1.4*	0.002
GPx (U/mL)	50 ± 2.8	20 ± 1.1*	0.004
Nitric oxide (µmol/L)	22.2 ± 9.7	49.6 ± 2.6*	0.04
SOD (U/ml)	27.3 ± 1.4	64 ± 2*	0.001
TAC (mM/L)	54.3 ± 2.3	24.6 ± 2.3*	0.001
MDA (nmol/mL)	10.9 ± 3.8	30.6 ± 1.2*	0.02

*TNF-α* Tumor necrosis factor-alpha, *GPx* Glutathione peroxidase, *SOD* Super oxide dismutase, *TAC* Total antioxidant capacity, *MDA* Malondialdhyde.

*Statistically significant when *P* < 0.05.

### Correlation between ultrasonographic and Doppler findings, gene expression pattern and serum profile of immune, APPs and antioxidant markers

The uterine horn diameter was positively correlated with the mRNA levels of CATand GPXand serum level of Cp (r = 1, *P* = 0.014, r = 1, *P* = 0.012 and r = 0.997, *P* = 0.049) respectively. Uterine artery diameter was positively correlated with the mRNA levels of A2M and FCAMR (r = 1, *P *= 0.016 and r = 0.998, *P* = 0.038) respectively and negatively correlated with mRNA levels of MAPK14 (r = − 998, *P* = 0.035). The endometrium thickness was positively correlated with serum level of Cp and IL10 (r = 1, *P* = 0.019 and r = 0.999, *P* = 0.032), respectively and negatively correlated with serum level of SAA (r = − 999, *P* = 0.023). Pulsatility index (PI) was positively correlated with the mRNA levels of MAP3K4, iNOS and CCl2 (r = 1, *P* = 0.019, r = 1, *P* = 0.015 and r = 0.999, *P* = 0.031) respectively and negatively correlated with mRNA levels of KCNT2 (r = − 998, *P* = 0.045). Resistance index (RI) was positively correlated with the mRNA levels of MAPK14 (r = 0.998, *P* = 0.035) and negatively correlated with mRNA levels of A2M and FCAMR (r = − 1, *P* = 0.016 and r = − 0.998, *P* = 0.038), respectively. Time average mean velocity (TAMEAN) was negatively correlated with mRNA levels of GPX(r = − 999, *P* = 0.024) and positively correlated with serum level of Cp (r = 1, *P* = 0.013).Blood flow volume‐ TAMAX was positively correlated with the mRNA levels of TGF (r = 0.998, *P* = 0.039). Blood flow volume‐ TAMEAN was negatively correlated with serum level of TNF-α (r = − 997, *P* = 0.049). The serum level of MDA was positively correlated with mRNA levels of ADAMTS20 and RXFP1 (r = 0.999, *P* = 0.031 and r = 0.998, *P* = 0.042), respectively. The serum level of TNF-α was positively correlated with mRNA levels of CAT and GPX (r = 1, *P* = 0.008 and r = 0.999, *P* = 0.034), respectively.

## Discussion

The objectives of the study were to validate the use Doppler ultrasonographic scan, gene expression and serum profile of metabolic, immune, APPs and antioxidant markers as diagnostic criteria for clinical endometritis in Egyptian Buffalo–Cows. Postpartum uterine infection is one of the most important disorders in bovines^6^. It causes great economic losses due to its negative effect on reproductive performance as it increases services per conception, the calving to first service interval and the calving to conception interval, reduces the risk of pregnancy, and decreases the conception rate^28^.

The significant increase of body temperature, pulse and respiratory rates in endometritis group could be attributed to interaction between the host immune system and bacterial endotoxins which trigger the cascade of events that lead to elevated temperature. Our clinical findings were similar to that observed by^29^ in cows.

The statistical examination of cervical diameter, endometrium thickness, and uterine horn diameter showed that endometritis buffalo–cow levels were significantly higher than normal levels (*p* > 0.05). Our findings were consistent with those reported by^30^, but in contrast to findings of^31^ in dairy cow. The later authors reported non-significant difference of cervical diameter, endometrium thickness, and uterine horn diameter between normal and endometritis animals. During uterine inflammation, where minimum penetration is necessary^32^, color Doppler method is a valuable tool for visualizing physiological and pathological processes of the reproductive system in cattle^33^. A greater PI denotes less perfusion to distant tissues, whereas a lower RI implies increased perfusion to the specific organ^34^. Our results were comparable to those in dairy cows published by^35^, But way from that study^30^, found no changes between groups in their spectral Doppler analysis of the uterine arteries. According to^36^, the bacterial infection in women leads to hyperaemia in pelvic organs, increasing uterine blood flow but causing low PI and RI values in the uterine artery. Clearance of infection leads to increased PI and RI values^37^. Studies by^35^, showed a negative correlation between RI and velocity and volume of blood flow. In the same context^38^, found that, TAMEAN and TAMAX were having an inverse relationship with RI and PI, while^35^ found non-significantly higher uterine artery diameter.

Through measuring the mRNA levels of immune (*A2M*, *TLR2*, *TGF-β*, *IRAK3*, *CCl2*, *FCAMR,* and *iNOS*), metabolic (*ADAMTS20*, *KCNT2*, *MAP3K4*, *MAPK14*, *RXFP1*, *FKBP5*, *RXFP1,* and* EPHA4*), and antioxidant (*SOD3*, *CAT*, *GPX*, and *NDUFS5*) genes, we examined the changes in the immune, metabolic, and antioxidant state in postparturient endometritis-affected buffalo cows compared with healthy ones. Gene expression levels were considerably higher in endometritis-affected buffaloes than in resistant ones for the genes *A2M*, *TLR2*, *IRAK3*, *CCl2*, *FCAMR*, *iNOS*, *ADAMTS20*, *KCNT2*, *MAP3K4*, *MAPK14*, *FKBP5,* and* EPHA4*. *The RXFP1*, *NDUFS5*, *TGF-β*, *SOD3*, *CAT,* and *GPX* genes were expressed at substantially lower levels in endometritis-affected buffaloes.

This is the first study to fully analyze the transcript levels of the immune, metabolic, and antioxidant indicators linked to the hazard of buffalo endometritis. Consequently, qualitative and quantitative differences in the investigated genes’ expression precede the development of bovine uterine disease. Greater relative quantities of mRNA for the *IL1A*, *IL6*, *IL17A*, *TNF*, *PGES*, and *PGHS2* genes were found in primiparous Holstein cows postpartum when compared to healthy cows^39^. Additionally, C3, C2, LTF, PF4, and TRAPPC13 had unique mRNA expression patterns in the blood and endometrial tissue of dairy cows with subclinical endometritis^40^. In contrast to control cows, cows with clinical and subclinical endometritis displayed a significant change in the mRNA expression of uterus-associated proinflammatory markers, according to^41^. In the endometrium of repeat breeding cows with and without subclinical endometritis, there were significantly more transcript levels of tumor necrosis factor and inducible nitric oxide synthase^42^. Three examined cytokines, including IL-1, IL-1β, and IL-6, were found to have increased gene expression in buffaloes with endometritis compared to healthy animals^43^. IL10, ATOX1, and GST genes were expressed at substantially lower levels with higher level of genes TLR4, TLR7, TNF-α, NCF4, LITAF, OXSR1, TKT, RPIA, and AMPD1 in endometritis-affected cows as compared with resistant ones^44^. Buffaloes were significantly more likely to express the inflammatory (IKBKG, LGALS, IL1B, CCL2, RANTES, MASP2, HMGB1, and S-LZ) genes when they had inflammatory reproductive diseases^45^.

The immune (*A2M*, *TLR2*, *TGF-β*, *IRAK3*, *CCl2*, and *iNOS*), metabolic (*ADAMTS20*, *KCNT2*, *MAP3K4*, *MAPK14*, *RXFP1*, *FKBP5, RXFP1 FCAMR,* and* EPHA4*), and antioxidant (*SOD3*, *CAT*, *GPX*, and *NDUFS5*) genes in endometritis-affected and healthy Holstein dairy cows were characterized in this research using a PCR-DNA sequencing technique. The findings show that the SNPs involving both categories vary. It is important to emphasize that the polymorphisms found and made available in this context provide additional data for the evaluated indicators when compared to the corresponding datasets acquired from GenBank. There have been recent studies targeting novel genes specific to livestock endometritis susceptibility using genome-wide association analysis^46,47^, but up to this point, no studies have examined the link between the SNPs in these genes and endometritis risk. The *Bubalus bubalis* gene sequences used in our study, which were reported in PubMed, are the first to demonstrate this association.

According to our knowledge, there has not been any prior research on the variation of the immune (*A2M*, *TLR2*, *TGF-β*, *IRAK3*, *CCl2*, and *iNOS*), metabolic (*ADAMTS20*, *KCNT2*, *MAP3K4*, *MAPK14*, *RXFP1*, *FKBP5*, *RXFP1 FCAMR,* and* EPHA4*), and antioxidant (*SOD3*, *CAT, GPX*, and *NDUFS5*) markers and how they relate to postparturient endometritis in buffaloes. The candidate gene method, however, was employed to keep track of the soundness of endometritis-affected livestock. For example, endometritis and *CXCR1* SNPs have been linked in Holstein dairy cows^48^. In dairy cattle, uterine infection was linked to lactoferrin (LTF) gene polymorphism^49^. There has also been evidence linking the beta defensin gene polymorphism and clinical endometritis in dairy cows^50^. SNPs in the *TLR4* and *TLR2* genes and endometritis tolerance in buffalo have been elaborated^51^. Nucleotide sequence variations between healthy and endometritis-affected cows were revealed using PCR-DNA sequencing for immune (TLR4, TLR7, TNF-α, IL10, NCF4, and LITAF), antioxidant (ATOX1, GST, and OXSR1), and erythritol-related (TKT, RPIA, and AMPD1) genes were reported by^44^. The immunological (IKBKG, LGALS, IL1B, CCL2, RANTES, MASP2, HMGB1, and S-LZ) genes’ nucleotide sequence differences between healthy buffaloes and buffaloes affected by inflammatory reproductive diseases were found by employing PCR-DNA sequencing^45^.

The alpha-macroglobulin (aM) family of proteins, which includes C3, C4, and C5, also includes alpha-2-macroglobulin (A2M)^52^. Additionally, it promotes the growth of macrophages and T cells^53^. Mutations in A2M contributed to mastitis susceptibility in dairy cows^54^. Innate immune systems, particularly Toll-like receptors and antimicrobial peptides, are vital for the endometrium’s first defense in contradiction of microorganisms^55^.

Transforming growth factor-beta (TGF-β) is a multifunctional peptide, belonging to a family of cytokines present in many cell types, involved in regulating proliferation, differentiation, adhesion, migration, and immune regulation^56^. Interleukin 1 receptor associated kinase 3 (IRAK3) is mainly found in monocytes and macrophages, hence it is also known as IRAK-M^57^. Thus, IRAK3 plays a crucial role in modulating TLR signalling pathways of innate immunity.

C–C Motif Chemokine Ligand 2 (CCL2), which encodes two tiny proteins that bind to the CCR2 receptors present on the surface of monocytes and neutrophils, respectively, acts as a potent chemokine for these cells^58^. The relationship between single nucleotide polymorphisms in the bovine CCL2 gene and production and health was investigated using Canadian Holstein cattle^59^. Nitric oxide (NO) plays a vital role in many physiological and pathological processes, and it is synthesized from the amino acid L-arginine by NO synthase (NOS). iNOS is encoded by the *NOS2A* gene, which is under the transcriptional control of inflammatory mediators produced by immunocompetent cells such as macrophages and neutrophils^60^.

The role of ADAMTS family proteases in reproductive function and disorders in humans is well known and mutations in ADAMTS20 were associated with endometrial tissue remodeling and inflammation^61^. A promising candidate gene is the KCNT2 gene on BTA 16 in cattle^62^ identified KCNT2 as a candidate gene for endometritis within 150 d after calving in first-parity Canadian Holstein cows. Recently^63^, applied a genome wide association analysis (GWAS) and fine mapping study for disease traits and identified the KCNT2 gene as a main candidate for ketosis in dairy cattle.

Inflammatory diseases may be treated by targeting mitogen-activated protein kinases (MAPKs), which are typically activated in response to inflammatory cytokines and cellular stress^64^. FKBP5 as a candidate gene interacting with polychlorinated biphenyls, which are increased in human endometritis^65^. Interestingly, FCAMR may function in immune response to microbes mediated by IgA and IgM^66^ showed that IgA, IgM, and IgG are the major immunoglobulins for blocking bacterial pathogens from adhering mucosal surface in the uterus. RXFP1 encodes a member of the leucine-rich repeat-containing subgroup of the G protein-coupled 7-transmembrane receptor superfamily. The encoded protein plays a critical role in pregnancy and parturition as a receptor for the protein hormone relaxin.

Erythropoietin-producing hepatocellular receptor A4 (*EphA4*) gene, as a crucial member of Eph–Ephrin family, encodes a pleiotropic cytokine and EphA4 protein is normally produced by the endometrium^67^.

Multi-pathogen bacterial infections of the vaginal tract develop in dairy cattle following urination^68^. A bacterial infection of the endometrium causes the production of chemokines and cytokines, which activates an inflammatory response. Leucocyte recruitment during inflammation has been reported to be interceded by inflammatory cytokines and complement fragments^69^. Endometritis is also characterized by unchecked extended inflammation linked to tissue damage, which causes the release of molecular forms accompanying injury, further aggravating inflammation and guaranteeing its perseverance^47^. Afterwards, oxidative stress is brought on by the extreme gathering of ROS^70^. These modifications are also associated with increased expression of molecules involved in LPS signaling, tissue remodeling, and acute phase response^40^. The aforementioned reasons could account for the significant amendment in the expression configuration of immune (*A2M*, *TLR2*, *TGF-β*, *IRAK3*, *CCl2*, and *iNOS*), metabolic (*ADAMTS20*, *KCNT2*, *MAP3K4*, *MAPK14*, *RXFP1, FKBP5*, *RXFP1 FCAMR,* and* EPHA4*), and antioxidant (*SOD3*, *CAT*, *GPX*, and *NDUFS5*) indicators in endometritis-affected buffaloes. Thus, we assume that an infectious etiology is to blame for the bovine endometritis in the study’s buffaloes. The endometritis-affected buffaloes were exhibiting a substantial inflammatory response, as shown by our real-time PCR data. Gene expression disruption can be used to characterize the common pathological processes, whereas normal gene expression controls the bulk of physiological mechanisms^71^.

The current study found that buffalo cows with clinical endometritis had normocytic hypochromic anemia, which was demonstrated by a substantial reduction in RBCs, Hb, PCV, neutrophilia, and monocytosis. Buffalo–cows undergo unanticipated nutritional and hormonal changes during the transition phase, which compromises their immune system^72^. The buffalo–cows are more susceptible to uterine infection as a result of this impaired immune response^73^. Since the MDA level in these buffalo cows was greater than it was in healthy buffalo cows, it is likely that the large increases in (TLC) and granulocytes were caused by elevated cortisol levels^74^. In buffalo–cows with endometritis, the TLC, granulocytes, and monocytes continued to rise, suggesting a possible prolonged peripheral inflammatory response in the buffalo–cows with uterine infection^74^. Bacterial toxins that are circulating in the circulation cause red blood cells to have certain shape abnormalities that cause them to become caught in the spleen network and cause regenerative anemia in animals. The immune system is also stimulated by the presence of germs to create more phagocytic cells, such as neutrophils and monocytes^75^. Our results disagreed with those published by^76^ in Egyptian Buffalo–Cows and Murrah buffaloes and^77^ in crossbreed cows.

APR (acute phase reaction) is one of the many systemic reactions that happen after an infection, damage, or even when the usual physiological balance changes. APR is a series of internal responses to inflammation that are mostly controlled by cytokines, which are produced by macrophages or other inflammatory cells. Production of pro-inflammatory cytokines (mainly IL-1β, TNF-α, and IL-6) at the site of injury, subsequently stimulates the production of APPs from a local site or at the liver. Several writers examined the use of APPs to assess domestic animal health status^78^. When compared to healthy buffalo–cows, there was a significant rise in the blood levels of Hp, SAA, Cp, IL-6, IL-10, and TNF- in the current study. Our results agreed with those provided by^79–81^.

In the current investigation, buffalo's cows with endometritis showed disrupted oxidative state, with significantly higher MDA and NO levels and lower activity of CAT, GPx, SOD, and TAC values than in healthy animals. It is well knowledge that inflammatory disorders are linked to heightened oxidative responses and diminished antioxidant defenses^82^. Reactive oxygen species (ROS), which damage DNA and cell membranes during infection, are produced at high levels in animals with clinical endometritis, as indicated by increased MDA levels^83^. The inflammation of uterine tissue is associated with increase the serum level of NO resulting in relaxation of smooth muscle and accumulation of inflammatory products in the uterus playing an important role in the increased severity of infection of the uterus. The higher NO level in blood is guessed as an inflammatory reaction of uterine tissue. Elevated values of serum nitric oxide causing loosening of smooth muscle and gathering of inflammatory consequences in the uterus assuming a significant part in the expanded seriousness of infection of the uterus^84^. The decrease in the concentration of antioxidant enzymes suggests that antioxidant enzymes are faithfully involved in the neutralization and scavenging of free radicals generated during oxidative stress. Reduced levels of antioxidant enzymes are thought to be due to the enzymes' role in converting harmful free radicals into harmless molecules. Our outcomes were like that detailed by^76,79,80,85,86^.

## Conclusion

This study confirmed that non-invasive transrectal Doppler ultrasound can be a useful tool to assess hemodynamic changes during uterine inflammation. Reference values ​​were also provided for further studies on blood flow in endometritis in Egyptian buffaloes. This technique also has great potential in the field of buffalo breeding to determine future fertility and complications during pregnancy. Our findings highlight the significance of SNPs in investigated immune and antioxidant genes as genetic markers and predisposing factors for endometritis resistance/susceptibility. These findings suggest that variability in these genes could be used as proxy biomarkers for such disorder in Egyptian-buffalo cow. The variable expression pattern of immune and antioxidant genes in resistant and non-resistant buffalo–cow to endometritis could be a reference guide and a biomarker that can be used to follow up health status of buffalo–cow. These data open a promising opportunity for limiting endometritis through selective breeding of animals based on genetic markers associated with natural resistance to infection.

## Materials and methods

### Animals and study design

A total number of 50 Egyptian buffaloes cows with an average of 7–12 years (mean ± SD: 9.42 ± 1.8) and a range of body weight 550–650 kg (mean ± SD: 600 ± 40.82) were used in this study. The experiment was carried out at Siwa Oasis, Egypt which lies between longitudes (Lat: 29° 06″ 29° 24″ N Long: 25° 16″ 26° 12″ E), and located 330 km southwest of the Mediterranean shoreline and at 65 km east of the Libyan borders and in Animal Reproduction Research Institute. Animals were housed in barns, both water and grasses were offered ad lib, with nearly 3 kg/day of commercial concentrate was offered for each buffalo cow. The examined animals were 25 cycling healthy buffalo–cow and 25 buffalo–cow with endometritis. The investigated buffalo–cows were subjected to through clinical examination including recording of temperature, pulse and respiratory rates^87^. Clinical endometritic was diagnosed as expulsed purulent (> 50% pus) uterine discharge detectable in the vagina more than 21 days after calving or muco-purulent (50% pus–50% mucus) uterine discharge detectable in the vagina after 26 days after calving. Estrus was synchronized through application of Ovisynch protocol as follow: on day zero the buffallo-cow received 100 µg GnRH in form of Gonadroline (Gonavet®,Veyx,Germany), on the 7th day the same buffalo–cows received 500 µg PGF2α in form of Cloprostenol (PGF Veyx Forte®,Veyx,Germany), on the 9th day 100 µg of GnRH (Gonavet®) was injected to the same buffalo–cows. All hormones injections were administrated I.M.

### Blood sampling

Ten milliliters of blood was collected from each buffalo–cow via jugular venipuncture at 8 O’clock morning. The collected blood was added to plain tubes (i.e., without anticoagulants) and to others containing EDTA to yield serum or whole blood, respectively. All samples were cooled on crushed ice and were transported immediately to the laboratory for further processing. Tubes containing whole blood were used for CBC and RNA extraction while those in plain tubes were kept overnight at room temperature and centrifuged at 3000 rpm for 15 min. Only clear sera were collected then aliquoted and kept frozen at  − 20 °C for subsequent biochemical analyses of energetic and oxidative stress markers.

### Ultrasonography and Doppler mode

**Figure 6:**
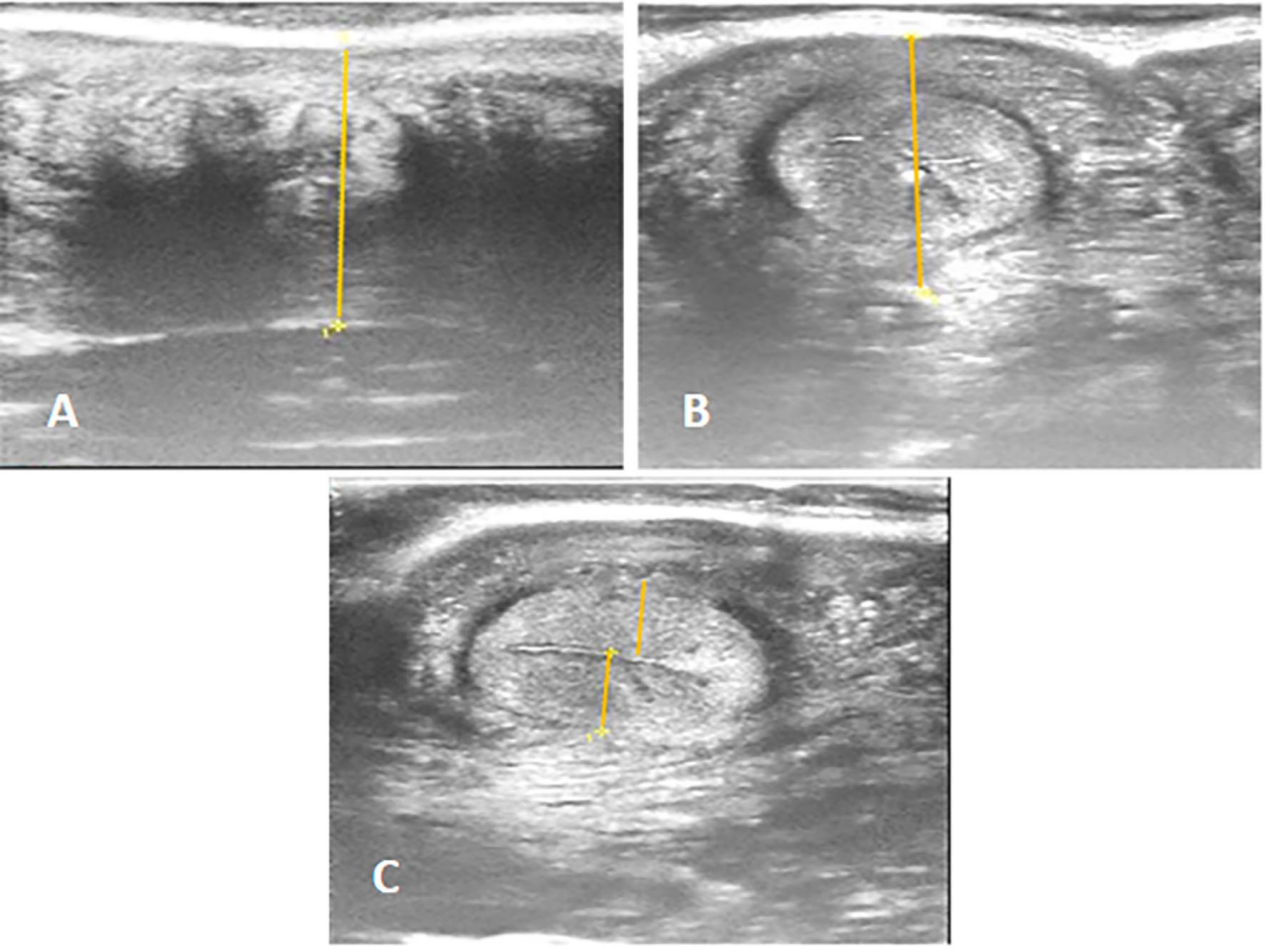
Transrectal ultrasonography, (**A**): Cervical diameter, (**B**): Uterine horn diameter, (**C**): Uterine thickness.

All animals were reproductively assessed by the same operator using ultrasound machine (Sonoscape, E1 Expert, China) provided with high frequency linear transducer: L741, 5–15 MHz to record the diameter of the cervix and uterine horn as well as endometrial thickness (Fig. 6A–C) according to^88^. The gain, brightness and contrast were set in optimal range for each examination.

All animals were enrolled for quantitative analysis of blood flow through the middle uterine artery (MUA), a branch of the internal iliac artery that is situated cranial to the external iliac artery and can be found in the mesometrium as a movable arterial vessel and easily seen by the color Doppler technique after the conventional ultrasonographic evaluation^35,89^. A high frequency linear transducer with a filter of 100 Hz, power of 90%, pulse repetition frequency (PRF) of 6 HZ, and a Doppler angel ranging between 0° and 60° was used to perform the Doppler assessment. The diameter (D; cm) of middle uterine arteries was measured in B-mode, and the mean of three measurements of vessel diameter per examination was calculated from a frozen, two-dimensional, greyscale image were used for statistical analysis. Blood flow indices including pulsatility index (PI), resistance index (RI), TAMEAN (Time average mean velocity) and the time average maximum velocity (TAMAX, cm/s).

Blood flow volume in mL/min was calculated using the equation^90^**:**

$$ {\text{Blood flow volume - TAMAX}} = {\text{TAMAX}} \times \pi \times \left( {{\text{D }} \times \, 0.{1}/{2}} \right)^{{2}} \times {6}0 $$

$$ {\text{Blood flow volume}}{ - }{\text{TAMEAN}} = {\text{TAMEAN}} \times \pi \times \left( {{\text{D}} \times 0.{1}/{2}} \right)^{{2}} \times {6}0 $$

### Total RNA extraction, reverse transcription and quantitative real time PCR

Total RNA was extracted from buffalo blood using Trizol reagent following the manufacturer instructions (RNeasy Mini Ki, Catalogue no.74104). The amount of extracted RNA was quantified and qualified using NanoDrop® ND-1000 Spectrophotometer. The cDNA of each sample was synthesized following the manufacture protocol (Thermo Fisher, Catalog no, EP0441). The gene expression pattern for coding fragments of genes encoding immune (*A2M*, *TLR2*, *TGF-β, IRAK3*, *CCl2*, *FCAMR,* and *iNOS*), metabolic (*ADAMTS20*, *KCNT2*, *MAP3K4*, *MAPK14*, *RXFP1*, *FKBP5*, *RXFP1,* and* EPHA4*), and antioxidant (*SOD3*, *CAT*, *GPX*, and *NDUFS5*) was assessed using quantitative RT-PCR using SYBR Green PCR Master Mix (2 × SensiFastTM SYBR, Bioline, CAT No: Bio-98002). Relative quantification of mRNA level was performed by real-time PCR using SYBR Green PCR Master Mix (Quantitect SYBR green PCR kit, Catalog no, 204141). Primer sequences were designed according to the PubMed published sequence of *Bubalus bubalis* (Table 6). The housekeeping gene *ß. actin* was used as a constitutive control for normalization. The reaction mixture was carried out in a total volume of 25 µl consisted of total RNA 3 µl, 4 µl 5 × Trans Amp buffer, 0.25 µl reverse transcriptase, 0.5 µl of each primer, 12.5 µl 2 × Quantitect SYBR green PCR master mix and 8.25 µl RNase free water. The final reaction mixture was placed in a thermal cycler and the following program was carried out: reverse transcription at 50 °C for 30 min, primary denaturation at 94 °C for 10 min followed by 40 cycles of 94 °C for 15 s, annealing temperatures for 1 min as shown in Table 6, and 72 °C for 30 s. At the end of the amplification phase, a melting curve analysis was performed to confirm the specificity of the PCR product. The relative expression of each gene per sample in comparison with *ß. actin* gene was carried out and calculated according to the 2^−ΔΔCt^ method^91^.

**Table 6:** Oligonucleotide primers sequence, accession number, annealing temperature and PCR product size of immune, metabolic, and antioxidant genes used in real time PCR.

Gene	Primer	Product length (bp)	Annealing temperature (°C)	Accession number	Source
A2M	F5′-TGTGCTGTGGTCCGTTTTTACC-3′	304	58	KF928932.1	Current study
	R5′-CGAGAATATGGGATGGGAGAA-3′				
TLR2	F5′-GCCTCTCATCAGGCTTCTTC-3′	224	56	NM_001290891.1	Current study
	R5′- GAATCTTCCTCCACTGTGTGA-3′				
TGF-β	F5′-CTGTGGTTGCTAATGCTGACG-3′	420	58	JQ995536.1	Current study
	R5′-CAGCTCTGCCCGAGAGAGCAAC-3′				
IRAK3	F5′-TGGCCGTCGTCGTGCTAGCAG-3′	310	60	XM_006066455.3	Current study
	R5′-CAATGTGACGAACATCCAGC-3′				
CCL2	F5′-CCAACAGCTTCCACGCTGAA-3′	356	58	HQ889748.1	Current study
	R5′-TGTGGAGTGAGTGCTCAAGGCT-3′				
FCAMR	F5′-GGACCTCGAACTTGCTGCAG-3′	330	60	XM_006043810.4	Current study
	R5′- CAGCTCCTGGTGGCTGCTGGC-3′				
iNOS	F5′-TGGTTCAAGGCATCCTTGAGCG-3′	445	60	EF562453.1	Current study
	R5′-CGGTACGTGAGCACGGCCACAG-3′				
ADAMTS20	F5′-TATACTTCCGCTACAGGGCACG-3′	369	56	XM_006077068.4	Current study
	R5′- TCACGTAGTGTTGAAGCCTGGC-3′				
KCNT2	F5′-CTCCATTCGTTGTCTCACGT-3′	420	58	XM_044943223.2	Current study
	R5′- TAACTGAGATAACCAAGCAGTA-3′				
MAP3K4	F5′-ATCAGACATTGGCTGGCCGGT-3′	450	60	XM_055536148.1	Current study
	R5′- ACTGGACCGTCGCTCTTAACA-3′				
MAPK14	F5′-TGACAGGCTATGTGGCCACCA-3′	300	58	XM_006050391.4	Current study
	R5′-CCGGTTCGTCGTCAGGATCGTG-3′				
RXFP1	F5′-TAGTACTGATGAATAACGTCC-3′	360	58	XM_044930387.2	Current study
	R5′- AAGAGAGATTCATGAGAGGT-3′				
FKBP5	F5′-CAATGACTACTGATGAAGCTG-3′	345	56	XM_044930861.2	Current study
	R5′- GAGCCATATGCATATTCTGGT-3′				
EPHA4	F5′-ATAGAAGCGGCAGGAGCAGCG-3′	221	58	XM_044937879.2	Current study
	R5′- CATCCATAATACTCACTTCCT-3′				
SOD3	F5′-CCTGTGACCAGCTGGTGAGGT-3′	360	56	XM_006041480.4	Current study
	R5′-CCGGGTCGATGGCCGCCGCCTG-3′				
CAT	F5′-TGACCACTGGAGGCGGTAATC-3′	362	58	XM_044929272.2	Current study
	R5′- TCCAACAAGATCCCAATTAC-3′				
GPX	F5′- TCTTGTTCTTCAAGTCCGCG -3′	287	58	XM_006053253.3	Current study
	R5′- AAGCCGAGCACGACCAGGCC-3′				
NDUFS5	F5′-TACGGCAGGCCTCCTAGTCG-3′	328	58	XM_006080987.3	Current study
	R5′- CGCTGTCTCTTGATGGCATTCA- 3′				
ß. actin	F5′- GGAATCCTGCGGTATTCACGA-3′	222	60	NM_001290932.1	Current study
	R5′- CCGCCAATCCACACAGAGTA -3′				

*A2M* alpha-2-macroglobulin, *TLR2* Toll-like receptor 2, *TGF-β* Transforming growth factor beta, *IRAK3* Interleukin 1 receptor associated kinase 3, *CCL2* C–C Motif Chemokine Ligand 2, *FCAMR* Fc alpha and Mu receptor, i*NOS* Inducible nitric oxide synthase,* ADAMTS20* ADAM metallopeptidase with thrombospondin type 1 motif 20, *KCNT2* Potassium sodium-activated channel subfamily T member 2, *MAP3K4* Mitogen-activated protein kinase kinase kinase 4, *MAPK14* Mitogen-activated protein kinase 14, *FKBP5* FKBP prolyl isomerase 5, *RXFP1* Relaxin family peptide receptor 1, *EPHA4* Ephrin type-A receptor 4, *SOD3* Superoxide dismutase 3, *CAT* Catalase, *GPX* Glutathione peroxidase, and *NDUFS5* NADH:ubiquinone oxidoreductase subunit s5.

### DNA sequencing and polymorphism detection

Before DNA sequencing, removing primer dimmers, nonspecific bands and other impurities was done. As described by^92^, purification of real time PCR products with expected size (target bands) was carried out using PCR purification kit following the manufacturer procedures (Jena Bioscience # pp-201 × s/Germany). Quantification of PCR product was carried out using Nanodrop (Uv–Vis spectrophotometer Q5000/USA) in order to yield high products and to ensure enough concentrations and purity of the PCR products^93^. To detect SNPs in genes investigated in control, and endometritis affected buffaloes, PCR products with target band were sent for DNA sequencing in forward and reverse directions using ABI 3730XL DNA sequencer (Applied Biosystem, USA), depending on enzymatic chain terminator technique developed by^94^.

Analysis of DNA sequencing data was carried out by chromas 1.45 and blast 2.0 software^95^. Differences were classified as single-nucleotide polymorphisms (SNPs) between PCR products of investigated genes and reference sequences available in GenBank. Based on data alignment of DNA sequencing, variation of amino acid sequence of the investigated genes between enrolled buffaloes was performed using the MEGA6 software package^96^.

### Biochemical analysis

The following commercial kits were used according to the standard protocol of the suppliers to quantify each of: serum amyliod A (SAA) using IBL International Crop (canda)® ELISA kits, haptoglobin (Hp) by Eagle Biosciences (Columbia) ELISA kits, caeruloplasmin (Cp) levels by Arbor Assays DetectX ® (USA)® kits. For malondialdehyde (MDA) (Biodiagnostic Egypt, CAT No: MD2529), catalase (CAT) (Biodiagnostic Egypt, CAT No: CA252417); glutathione peroxidase (GPx) (Biodiagnostic Egypt, CAT No: GR 2511), total antioxidant capacity (TAC) (Biodiagnostic Egypt, CAT No: TA25 13), nitric oxide (NO) (Biodiagnostic Egypt, CAT .No.NO2533), super oxide dismutase (SOD) (Biodiagnostic Egypt, CAT No: SD 25 20), IL 6 (BOSTER BIOLOGICAL TECHNOLOGY, CAT No: EK0412) and TNF-α ELISA Kit (AVIVA SYSTEM BIOLOGY); IL-10 (ELISA kits of Ray Biotech Company®).

### Statistical analysis

Statistical analyses were carried out using a statistical software program (SPSS, ver.20, Inc., Chicago, USA). Descriptive statistics were performed for all parameters. Student’s t-test was used analyze the data. Results were considered statistically significant at P ˂ 0.05.

### Ethical approval and informed consent

The experimental procedures were approved by the Experimental Animal Care Committee of Desert Research Center, Egypt (Approval No. 2022–0180), and all protocols were carried out in accordance with guidelines and regulations of the Universal Directive on the Protection of Animals Used for Scientific Purposes. All protocols follow the ARRIVE guidelines for reporting animal research (https://arriveguidelines.org).

## Data Availability

The datasets used and/or analyzed during the current study are available from the corresponding author upon reasonable request.

## Abbreviations

MUA: Middle uterine artey
PI: Pulsatility index
RI: Resistance index
TAMAX: The time average maximum velocity
TAMEAN: Time average mean velocity
RBC: Erythrocytes count
Hb: Hemoglobin
PCV: Packed cell volume
MCV: Mean corpuscular volume
MCH: Mean corpuscular hemoglobin
MCHC: Mean corpuscular hemoglobin concentration
WBC: Total leukocytes count
TNF-α: Tumor necrosis factor-alpha
GPx: Glutathione peroxidase
SOD: Super oxide dismutase
TAC: Total antioxidant capacity
MDA: Malondialdhyde.
A2M: Alpha-2-macroglobulin
TLR2: Toll-like receptor 2
TGF-β: Transforming growth factor beta
IRAK3: Interleukin 1 receptor associated kinase 3
CCL2: C–C motif chemokine ligand 2
FCAMR: Fc alpha and Mu receptor
iNOS: Inducible nitric oxide synthase
ADAMTS20: ADAM metallopeptidase with thrombospondin type 1 motif 20
KCNT2: Potassium sodium-activated channel subfamily T member 2
MAP3K4: Mitogen-activated protein kinase kinase kinase 4
MAPK14: Mitogen-activated protein kinase 14
FKBP5: FKBP prolyl isomerase 5
RXFP1: Relaxin family peptide receptor 1
EPHA4: Ephrin type-A receptor 4
SOD3: Superoxide dismutase 3
CAT: Catalase
GPX: Glutathione peroxidase
NDUFS5: NADH:ubiquinone oxidoreductase subunit s5
A: Alanine
D: Aspartic acid
E: Glutamic acid
F: Phenylalanine
G: Glycine
H: Histidine
I: Isoleucine
L: Leucine
M: Methionine
N: Asparagine
P: Proline
R: Argnine
S: Serine
T: Threonine
V: Valine
